# Molecular authentication and differentiation of *Dendrobium* species by rDNA ITS region sequence analysis

**DOI:** 10.1186/s13568-019-0767-8

**Published:** 2019-04-19

**Authors:** Hongmei Liu, Chengxin Fang, Tingmo Zhang, Li Guo, Qiang Ye

**Affiliations:** State Key Laboratory Breeding Base of Systematic Research, Development and Utilization of Chinese Medicine Resources, Chengdu University of Traditional Chinese Medicine, Chengdu, 611137 People’s Republic of China

**Keywords:** *Dendrobium*, rDNA ITS, Phylogenetic tree, Species identification, Molecular differentiation

## Abstract

Owing to their significant medicinal and edible values, the natural *Dendrobium* species have underdone over-collection and habitat destruction, and cultivated species emerged for candidates. However, these *Dendrobium* plants are similar in shape to be easily confused, leading to extreme difficulties for identification based on their morphological and chemical features. In this study, the rDNA ITS region sequence analysis was developed for rapid and accurate identification of thirteen wild and cultivated *Dendrobium* species belonging to two sections *Formosae* and *Chrysotoxae*. By cloning and sequencing the rDNA ITS region genes from 13 *Dendrobium* species, the phylogenetic relationships among them were analyzed. Results showed that the variation of the ITS region, together with the lengths and Guanine and Cytosine contents of ITS, 5.8s rDNA, ITS1 and ITS2 sequences occurred in the tested *Dendrobium* species, and which from section *Chrysotoxae* was higher than that from section *Formsae*. Phylogenetic analysis based on neighbor-joining and maximum p-arsimony trees indicated that the *Dendrobium* species of sections *Formosae* and *Chrysotoxae* could be well divided into two groups. A majority of *Dendrobium* species exhibited distinctive ITS2 secondary structures, while for those with close genetic relationships were similar. Therefore, the ITS2 region sequence analysis is simple, quick, and highly reliable that can be used as an effective tool for molecular identification and classification, as well as the reconstruction of the phylogeny of wild and cultivated *Dendrobium* species belonging to different sections.

## Introduction

The genus *Dendrobium* is one of the largest genera in the family *Orchidaceae* including approximately 1600 species (Chiang et al. 2012; Wu et al. 2009) that is mainly distributed in tropical and subtropical Asia, and northern and eastern Australia (World Checklist of Selected Plant Families 2015; Zhu et al. 2009). *Dendrobiums* species have been thought as one of the most valuable medicinal genus in folk medicine and industrial crop with excellent medicinal merits, such as clearing unhealthy heat, nourishing Yin, benefiting the stomach, enhancing the body’s immunity, resisting cancer, and prolonging life (Ge et al. 2018; Zha et al. 2017; Zhao et al. 2017). And they have long been extensively used as the popular tonic and traditional Chinese medicines (TCMs) in many Asian countries (Chinese Pharmacopoeia Committee 2015), as well as a high-quality health food in South and Southeast Asia for hundreds of years (Kanlayavattanakul et al. 2018; Zhu et al. 2009).

However, the slow growth rate and excessive harvesting of *Dendrobium* species has left the wild and natural sources critically endangered and looking for the proper substitutes is in urgency. In this case, three cultivated species including *D. nobile*, *D. chrysotoxum*, and *D. fimbriatum* are officially allowed for use (Chinese Pharmacopoeia Committee 2015). Whereas, motivated by the medicinal values and economic benefits, a few of other species, such as *D. officinale*, *D. longicorna*, *D. aphyllum*, *D. bellatulum*, etc., are also widely used in various herbal commodity markets or in global horticultural trade (Wu et al. 2009; Zeng and Hu 2004). In China, about 80 species of this genus and two varieties can be found on the market. And in the traditional product region, cultivated *Dendrobiums* have already become the major resource. Owing to different environmental and developmental factors during the plant and growth processes, the constituent compositions and their contents in these *Dendrobium* species vary significantly among the raw herbs, even if the same species from different locations (Liang et al. 2004). But, the similar appearance and tissue structure among these species have led to notorious difficulties to identify them. The drug markets of *Dendrobium* species from one place are always complex with those from other places (Niu et al. 2018; Xu et al. 2014). Therefore, it is critical and important to precisely differentiate the wild and cultivated *Dendrobiums* plants at specie level for guaranteeing their quality and safety in practical use, as well as genetic resource conservation.

Although some methods, such as gas chromatography–mass spectrometry fingerprint (Chen et al. 2016), fourier transform infrared spectroscopy (Chen et al. 2015), capillary electrophoresis (Zha et al. 2009) have been applied for the authentication of *Dendrobium* species, they are complicated needing professional operators, cost- and time-consuming, and hard to distinguish the commercial samples that share similar textures, chemical and microscopic characteristics after processing and preparation as decoction pieces. In recent decades, molecular techniques, such as DNA barcoding (Wilson et al. 2019) and real-time PCR (Sobrino-Gregorio et al. 2019) have been used to differentiate many closely related species with significant advantages in the fields of taxonomy, phylogeny, evolution and breeding (Teixeira da Silva et al. 2016), especially for the morphological analogues or complexes including *Dendrobium* species. Currently, nuclear sequences [including internal transcribed spacer (ITS), intergenic spacer (IGS)] and plastid genes (*matK*, *rbcL*, *psbA*-*trnH*, *trnL intron*, etc.) based multiple specific genomics and DNA barcoding method is mainly applied for the discernment of *Dendrobium* species (Xiang et al. 2013; Xu et al. 2014). Among them, the ribosomal DNA (rDNA) ITS regions exhibited relatively high mutation and evolution rates, and high authentication efficiency regarding the length and sequence, which have been the most frequently utilized barcodes for rapid identification of *Dendrobium* at the genus and species levels (Ye et al. 2014; Feng et al. 2015). However, from then on, the application of rDNA ITS region sequences for the differentiation of wild and cultivated *Dendrobium* species has not been reported.

So, based on our previous findings and the reported ITS sequence information of *Dendrobium* specie plants, this study attempted to first amplify ITS sequences from the plants by using the optimized extraction methods for DNA to obtain the rDNA ITS region sequences through gene cloning and sequencing, which were finally to be used for distinguishing and differentiating 13 wild and cultivated *Dendrobium* species of sections *Formosae* and *Chrysotoxae* in China. Our results have shown that rDNA ITS region sequences could be used as valuable markers for rapid and reliable authentication study of *Dendrobium* species.

## Materials and methods

### Plant materials

**Fig. 1:**
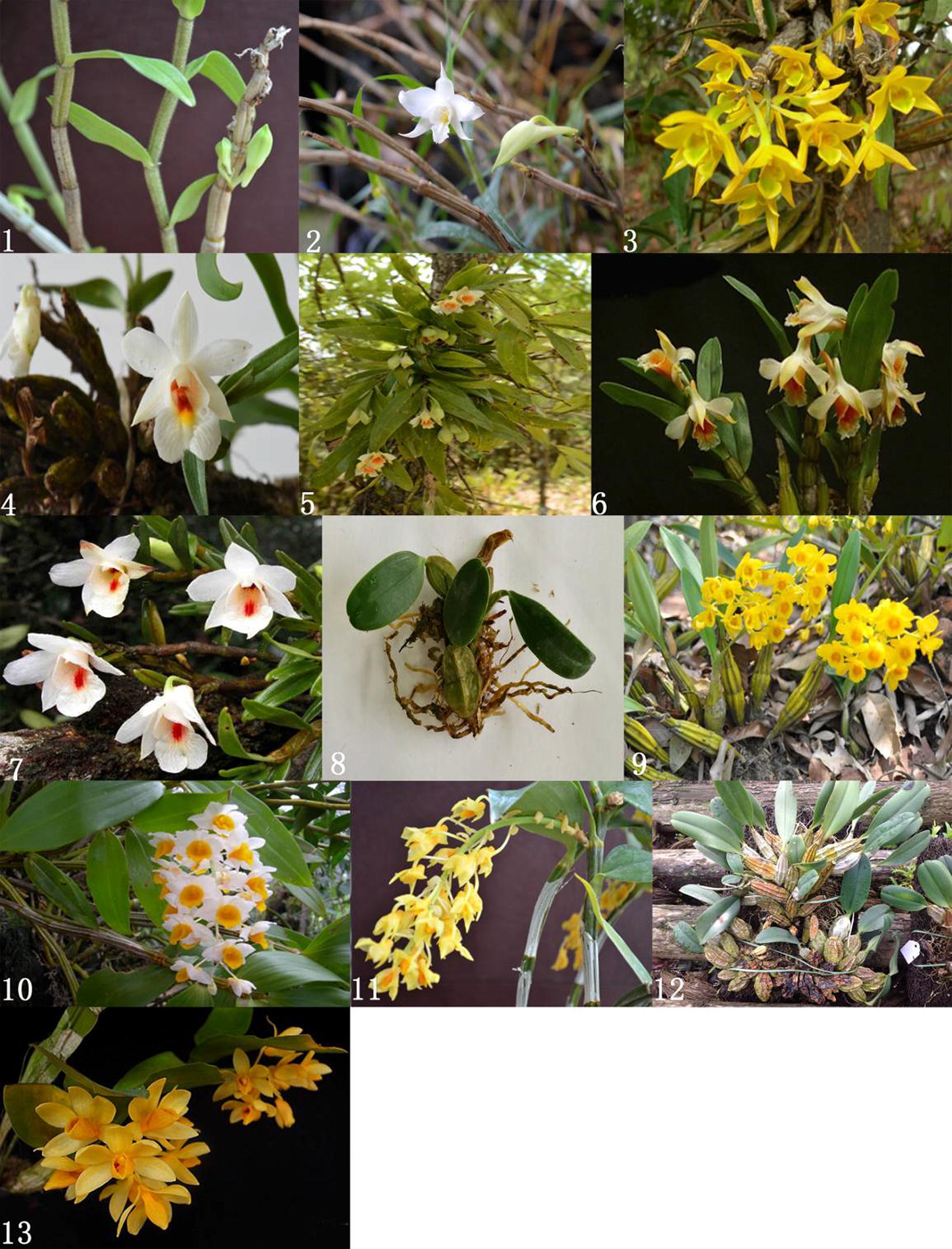
The 13 tested *Dendrobium* species. 1, *D. longicorna*; 2, *D. infundibulum*; 3, *D. trigonopus*; 4, *D. bellatulum*; 5, *D. williamsonii*; 6, *D. cariniferum*; 7, *D. sinense*; 8, *D. jenkinsii*; 9, *D. chrysotoxum*; 10, *D. thyrsiflorum*; 11, *D. densiflorum*; 12, *D. lindleyi*; 13, *D. sulcatum*

A total of 13 species of wild and cultivated *Dendrobium* plants were collected from the main distribution areas-Yunan province of China with Nos. 1–7 of section *Formosae* and Nos. 8–13 of section *Chrysotoxae*. The detailed information of these plants was listed in Table 1 with corresponding pictures in Fig. 1. The voucher samples were deposited in National Traditional Chinese medicine Germplasm Database of Chengdu University of Traditional Chinese medicine, Chengdu, China.

**Table 1:** *Dendrobium* specie plants used in the study

No.	Species	sources	Source county/city	Section	GenBank No.
1	*D. longicorna*	Cultivated	Yuannan, China	Sect.Formosae	MK522197
2	*D. infundibulum*	Cultivated	Yuannan, China	Sect.Formosae	MK522210
3	*D. trigonopus*	Wild	Yuannan, China	Sect.Formosae	MK522215
4	*D. bellatulum*	Cultivated	Yuannan, China	Sect.Formosae	MK522222
5	*D. williamsonii*	Wild	Yuannan, China	Sect.Formosae	MK522234
6	*D. cariniferum*	Cultivated	Yuannan, China	Sect.Formosae	MK522238
7	*D. sinense*	Wild	Yuannan, China	Sect.Formosae	MK522249
8	*D. jenkinsii*	Wild	Yuannan, China	Sect.Chrysotoxae	MK522193
9	*D. chrysotoxum*	Wild	Yuannan, China	Sect.Chrysotoxae	MK522232
10	*D. thyrsiflorum*	Wild	Yuannan, China	Sect.Chrysotoxae	MK522235
11	*D. densiflorum*	Cultivated	Yuannan, China	Sect.Chrysotoxae	MK522257
12	*D. lindleyi*	Wild	Yuannan, China	Sect.Chrysotoxae	MK522261
13	*D. sulcatum*	Cultivated	Yuannan, China	Sect.Chrysotoxae	MK522262

### DNA extraction

Approximately 100 mg flesh leaves of each sampled specimen were randomly collected for genomic DNA extraction using a CW0531A NuClean Plant Gen DNA Kit (Hefei, China) as the instructions of the manufacturer. DNA extraction from the 13 *Dendrobium* plants was performed using the two-step cetyltrimethylammonium bromide (CTAB) method. The DNA samples in the quality requirements (A260/280 ratio = 1.8–2.0, A260/230 ratio > 1.7, and DNA concentration > 100 ng/mL) were selected for sequencing. The obtained genomic DNA was stored at − 20 °C for further analysis.

### DNA amplification and sequencing

The sequences of the following pair of universal primers: P1, 5′-CGTAA CAAGGTTTCCGTAGGTGAAC-3′ and P2, 5′-TTATTGATATGCTTAAACTC AGCGGG-3′, that were synthetized by Tsing Ke Biotechnology Co., Ltd. (Chengdu, China), were used to amplify the rDNA ITS of *Dendrobium* plants. The following polymerase chain reaction (PCR) steps were conducted: The mixture containing 25 μL of 2 × Es Taq Master Mix polymerase, 2 μL of each primer, 1 μL of genomic DNA, and 20 μL of distilled water was denatured at 94 °C for 3 min, then it underwent 35 cycles of 40 s at 94 °C, 40 s at 56.4 °C, 1.5 min at 72 °C, and then a final extension for 5 min at 72 °C. The PCR products in TE (1.0%, *w/v*) were detected by using the agarose gel electrophoresis method for the concentration and quality of DNA, and were finally photographed under UV light exposure. The remaining PCR products were stored at − 20 °C until use. The amplified product was directly sequenced according to the dideoxy nucleotide chain termination method (Tsing Ke Biotechnology Co. Ltd., Chengdu, China).

### Data analysis

The DNA sequences were compared and aligned using MAGA 5.0. The genetic distance matrix was calculated by the two-parameter method. Phylogenetic trees were constructed by Neighbor-joining (NJ) and Maximum p-arsimony (MP) method with interior branch tests of 1000 replicates based on the alignments of ITS1, 5.8S rDNA, ITS2. The secondary structure of rDNA was predicted by using the bioinformatics software based on ITS2 sequence. (http://its2.bioapps.biozentrum.uni-wuerzburg.de/).

## Results

### PCR amplification and sequence characteristics

The sequencing characteristics were aligned with BLAST in National Center for Biotechnology Information (NCBI). All *Dendrobium* species can match with corresponding species by identity value of 99% or 100%. The ITS (ITS1-5.8S-ITS2) region from 13 *Dendrobium* species were aligned in Table 2.

**Table 2:** ITS and 5.8S rDNA lengths and GC contents of *Dendrobium* species

Species	Lengths (bp)				GC content (%)			
	ITS	5.8 s	ITS1	ITS2	ITS	5.8 s	ITS1	ITS2
*D. longicorna*	638	163	228	247	53.9	58.3	52.6	52.2
*D. infundibulum*	639	163	229	247	54.1	57.7	53.7	52.2
*D. trigonopus*	643	163	233	247	52.7	55.8	52.8	50.6
*D. bellatulum*	630	163	229	247	54.5	58.9	53.3	52.6
*D. williamsonii*	646	163	240	243	53.3	57.7	51.3	52.3
*D. cariniferum*	636	163	230	243	53.8	57.7	52.2	52.7
*D. sinense*	638	163	228	247	53.1	58.3	53.1	49.8
*D. jenkinsii*	643	163	232	248	50.9	57.7	47.4	49.6
*D. chrysotoxum*	651	163	243	245	54.8	58.9	51.9	55.1
*D. thyrsiflorum*	641	164	230	247	52.6	57.3	49.1	52.6
*D. densiflorum*	641	163	231	247	52.7	57.1	48.9	53.4
*D. lindleyi*	644	163	234	247	50.5	58.9	48.3	47.0
*D. sulcatum*	644	163	234	247	53.7	59.5	50.9	52.6

G + C, guanine and cytosine

As for the 7 *Dendrobium* plants of section *Formsae*: it could be found that the lengths of ITS sequence among the 13 *Dendrobium* species varied from 630 to 646 bp for the ITS region, from 228 to 240 bp for the ITS1 region, and from 243 to 247 bp for the ITS2 region, while was 163 bp for the 5.8s rDNA gene. The polymorphic numbers of variation sites were 119 bp (18.5%) in the ITS region, 65 bp (27.1%) in the ITS1 region, 8 bp (4.9%) in the 5.8s rDNA, and 46 bp (18.6%) in the ITS2 region. In addition, the contents of Guanine and Cytosine (GC) were from 52.7 to 54.5% in the ITS region, from 55.8 to 58.9% in the 5.8s rDNA, from 51.3 to 53.7% in the ITS1 region, and 49.8% to 52.7% in the ITS2 region. The average GC contents of ITS, ITS1, 5.8s rDNA and ITS2 were 53.6%, 52.7%, 57.8% and 51.8%, respectively.

While, for the 6 *Dendrobium* plants of section *Chrysotoxae*: It was shown that the lengths of ITS sequence were from 641 to 651 bp for the ITS region, from 163 to 164 bp for the 5.8s rDNA gene, from 230 to 243 bp for the ITS1, and from 245 to 248 bp for the ITS2 region. The numbers of the variation sites were 196 bp (30.1%) in the ITS region, 109 bp (44.9%) in the ITS1 region, 11 bp (6.7%) in the 5.8s rDNA, and 76 bp (30.6%) in the ITS2 region. In addition, the GC contents varied from 50.5 to 54.8% in the ITS region, 47.4% to 51.9% in the ITS1 region, 57.1% to 59.5% in the 5.8 s rDNA region, and 47.0% to 55.1% in the ITS2 region. And the average GC contents of ITS, ITS1, 5.8 s rDNA and ITS2 regions were 52.5%, 49.4%, 58.2% and 51.7%, respectively.

Then, it could be concluded that the polymorphic sites of 5.8s rDNA were less than that of ITS1 and ITS2, and the variation rates were very low. Previous studies also exhibited that 5.8s rDNA are highly conserved, and the ITS1 and ITS2 regions were more variable. Thus, the diversity of the ITS region could be used widely as a molecular marker for species authentication and polygenetic analysis. The lengths of the ITS2 region were a bit longer than that of the ITS1 region. The mutation rates of the ITS2 region were less than ITS1 region, which indicated that the diversity in the ITS1 region is much higher than that in the ITS2 region, and both the two regions would provide more molecular evidence for the accurate identification of *Dendrobium* species. In addition, the results showed that the variation from distinct groups of *Dendrobium* was different, and the variation rate of *Dendrobium* in section *Chrysotoxae* was higher than that from section *Formsae*.

In summary, the lengths and GC contents of ITS, 5.8s rDNA, ITS1 and ITS2 sequences from the collected *Dendrobium* species were relatively variable, which was satisfactory for next analysis.

### Genetic distance and phylogenetic analysis

With *Pholidota yunnanensis* obtained from NCBI as an out-group, the genetic distance matrix and phylogenetic tree were established based on ITS sequences (ITS1-5.8S-ITS2) of the 13 *Dendrobium* species and *Pholidota yunnanensis*, which were shown in Table 3. It could be observed that the genetic distances among the 14 species were within the range of 0.003–0.283 by the Kimura 2-parameter (K2P) model. Among the seven species of section *Formsae*, the range of genetic distances was from 0.003 to 0.135, and from 0.007 to 0.184 for the six *Dendrobium* species of section *Chrysotoxae*. *D. williamsonii* and *D. cariniferum* were relatively close with a genetic distance of 0.003. *D. chrysotoxum* and *D. lindleyi* were more divergent with a genetic distance of 0.184. *Pholidota yunnanensis*, as the out-group, exhibited the highest genetic distance of 0.227 to 0.283 and the largest differences from the 13 *Dendrobium* species. These findings were in agreement with the previous report (Tsai et al. 2004).

**Table 3:** Genetic distances of the ITS sequences among *Dendrobium* species (Nos. 1–13) and *Pholidota yunnanensis* (No. 14)

No.	1	2	3	4	5	6	7	8	9	10	11	12	13	14
1														
2	0.010													
3	0.128	0.123												
4	0.026	0.023	0.111											
5	0.050	0.047	0.116	0.037										
6	0.050	0.047	0.116	0.037	0.003									
7	0.020	0.016	0.135	0.032	0.052	0.052								
8	0.136	0.132	0.150	0.122	0.132	0.136	0.136							
9	0.153	0.149	0.169	0.135	0.151	0.151	0.157	0.175						
10	0.120	0.116	0.141	0.107	0.116	0.118	0.124	0.147	0.159					
11	0.118	0.114	0.139	0.105	0.114	0.116	0.122	0.143	0.157	0.007				
12	0.144	0.142	0.142	0.130	0.140	0.144	0.147	0.064	0.184	0.142	0.138			
13	0.124	0.122	0.146	0.111	0.120	0.122	0.126	0.149	0.157	0.098	0.096	0.154		
14	0.247	0.246	0.281	0.227	0.241	0.244	0.250	0.282	0.261	0.266	0.264	0.283	0.273	

1, *D. longicorna*; 2, *D. infundibulum*; 3, *D. trigonopus*; 4, *D. bellatulum*; 5, *D. williamsonii*; 6, *D. cariniferum*; 7, *D. sinense*; 8, *D. jenkinsii*; 9, *D. chrysotoxum*; 10, *D. thyrsiflorum*; 11, *D. densiflorum*; 12, *D. lindleyi*; 13, *D. sulcatum*; 14, *Pholidota yunnanensis*

**Fig. 2:**
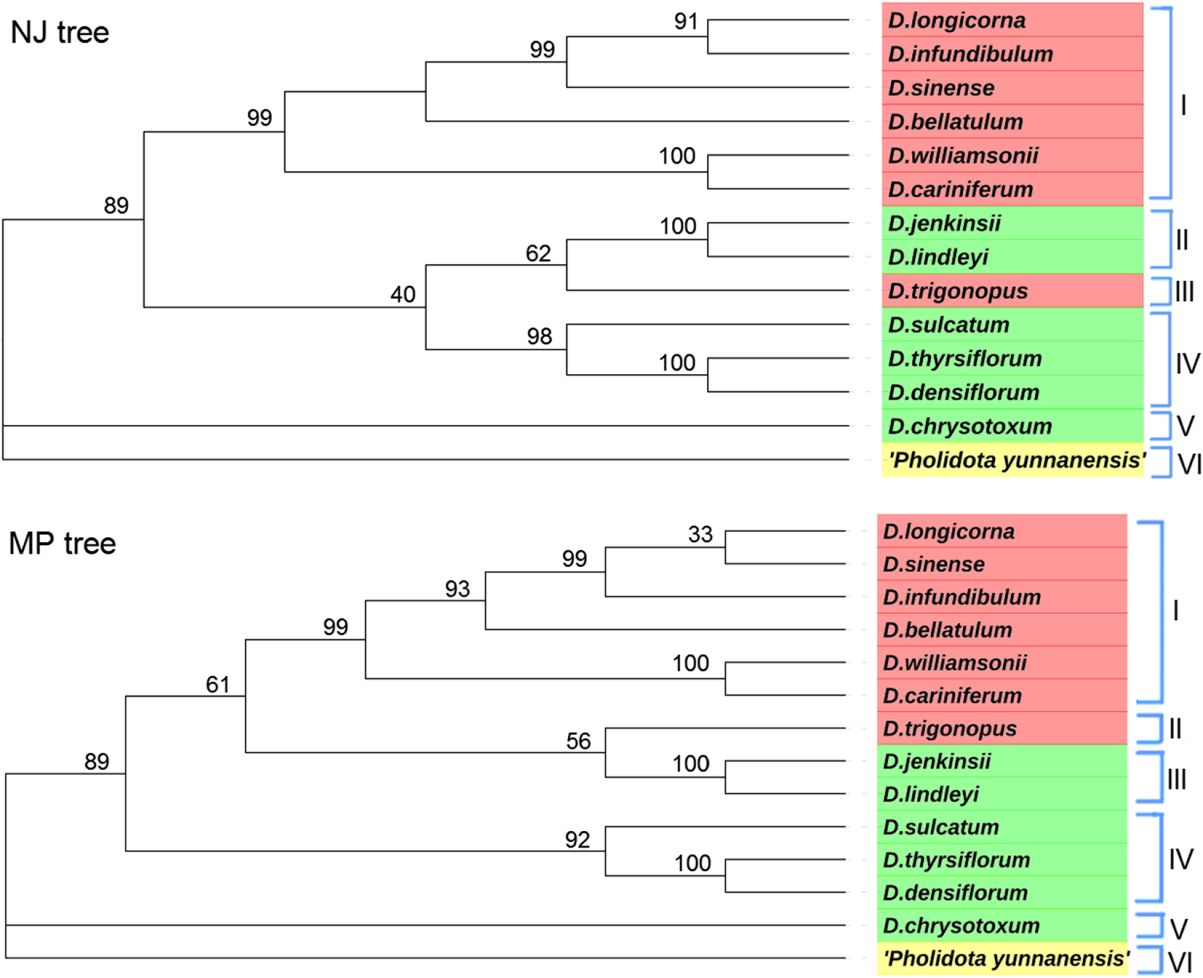
Neighbor joining (NJ) tree and Maximum parsimony (MP) tree generated using ITS (ITS1-5.8S-ITS2) sequences of *Dendrobium* species and *Pholidota yunnanensis*. (*D. longicorna*: MK522197; *D. infundibulum*: MK522210; *D. trigonopus*: MK522215; *D. bellatulum*: MK522222; *D. williamsonii*: MK522234; *D. cariniferum*: MK522238; *D. sinense*: MK522249; *D. jenkinsii*: MK522193, *D. chrysotoxum*: MK522232; *D. thyrsiflorum*: MK522235; *D. densiflorum*: MK522257; *D. lindleyi*: MK522261; *D. sulcatum*: MK522262)

Then, the phylogenetic trees were constructed by the NJ and MP methods, which were in agreement. It could be found from the NJ and MP trees in Fig. 2 that the 13 *Dendrobium* species could be clearly grouped in six clusters: *D. longicorna*, *D. infundibulum*, *D. sinense*, *D. bellatulum*, *D. williamsonii* and *D. cariniferum* that all belong to section *Formsae* were grouped together into cluster I with 99% similarity. However, *D. trigonopus* was separated in a single cluster (cluster III) from section *Formsae* species. The results were in accordance with previous researches (Xiang et al. 2013; Xu et al. 2014). These findings provided more evidences for the *Dendrobium* species in section *Formsae* as monophyletic except *D. trigonopu*, which showed that cluster analysis of section *Formsae* based on molecular characteristics was in match with the classification systems based on morphological analysis.

*D. jenkinsii* was grouped together with *D. lindleyi* into cluster II (NJ tree) and III (MP tree) with 100% similarity. *D. sulcatum*, *D. thyrsiflorum* and *D. densiflorum* were grouped together into cluster IV with 98% (NJ tree) and 92% (MP tree) similarity, respectively. Samples of the *Dendrobium* species in cluster II (NJ tree) or III (MP tree) and cluster IV belong to section *Chrysotoxae*. In addition, *D. chrysotoxum* was separated from the other 12 *Dendrobium* species with the interior branch test of 89%. From the NJ tree, the *Dendrobium* species of section *Chrysotoxae* except *D. chrysotoxum* were divided into three branches. Thus, it can be concluded that the phylogenetic tree for section *Chrysotoxae* species was not completely matched with the results of the classification systems based on morphological characters.

### Secondary structure of ITS2 sequences

**Fig. 3:**
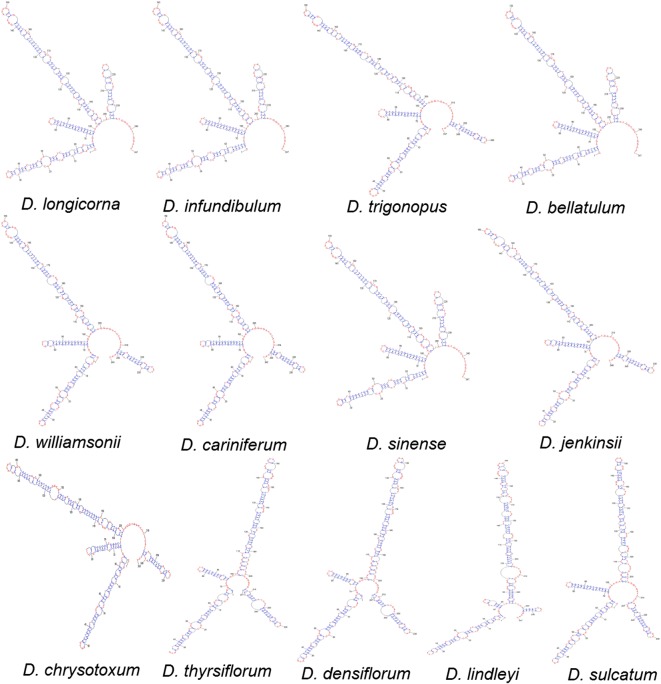
Diagram of secondary structure of ITS2 transcript of *Dendrobium* species

**Fig. 4:**
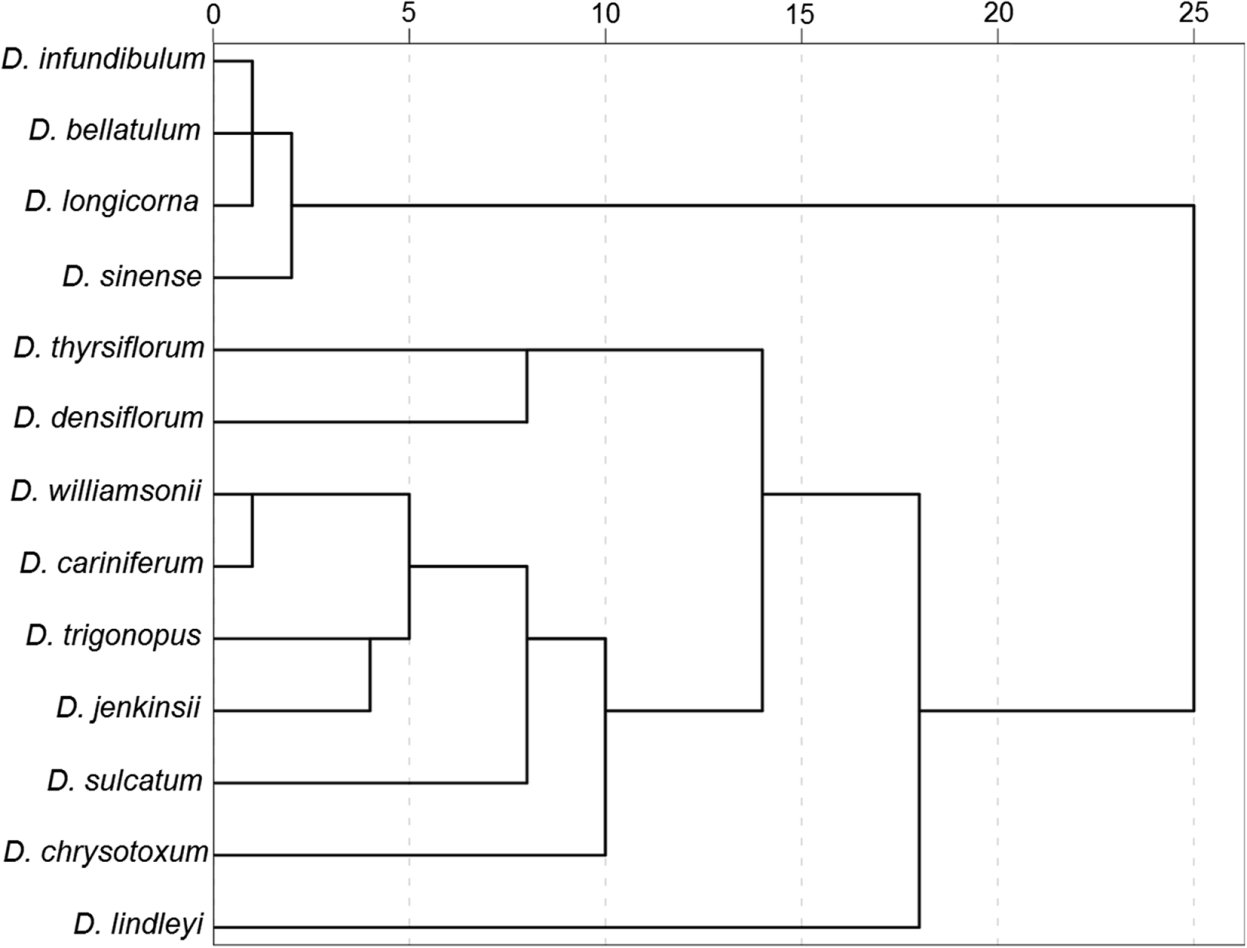
Dendrogram of hierarchical cluster based on the angle among Helix I, II, III and IV, the length of Helix I, II, III and IV, and the number of Loop in Helix I, II, III and IV

It is known that secondary structure is a guide to align the nucleotide positions of the ITS2 sequences with advantages of convenience and a wider range of taxonomy comparison (Coleman 2003). The secondary structure can be vividly regarded as a four-fingered hand and consists of Helix I, II, III and IV. The difference of secondary structure are mainly divided into the following three types: (1) the difference of angle among Helix I, II, III and IV; (2) the difference of length of Helix I, II, III and IV; and (3) the difference of the number and shape of Stem and Loop in Helix I, II, III and IV, which illustrate the morphological characteristics of ITS2 between species. Currently, all the secondary structures of genus *Dendrobium* have not been established. Hence, a secondary structure may have matched several *Dendrobium* species. Here, the secondary structures of the 13 *Dendrobium* species have obtained and were shown in Fig. 3. Then, based on the measured angle among Helix I, II, III and IV and the length of Helix I, II, III and IV was, as well as the counted number of Loop in Helix I, II, III and IV, the dendrogram of hierarchical cluster (Fig. 4) was constructed by means of between-groups linkage, and the distance was calculated by the Block method.

It could be seen that *D. longicorna*, *D. infundibulum*, *D. bellatulum* and *D. sinense* were in one cluster and shared the highest similarity based on the ITS2 secondary structure mode. This finding was supported by the previous result of a close genetic relationship among the four species based on the genetic distance analysis. The four *Dendrobium* species are clustered into a class with a relatively high bootstrap supporting rate in phylogenetic trees. *D. williamsonii* and *D. cariniferum* hold the same secondary structure, which also occurred between *D. thyrsiflorum* and *D. densiflorum*. The genetic distances between them were also extremely low and they were gathered together with a 100% bootstrap supporting rate in the phylogenetic trees. *D. trigonopus* and *D. jenkinsii* were clustered into the same group with far relationship in the phylogenetic trees. Thus, it could be concluded that the dendrogram based on secondary structure was partially matched with the results of phylogenetic tree. These studies indicated that the secondary structure of the ITS2 regions could enhanced the clustering patterns of the taxonomic levels, and sequence + structure based phylogenetic analysis was more reliable than single sequence analysis for the *Dendrobium* species regarding their molecular authentication and differentiation.

## Discussion

A rapid and accurate method for molecular identification and differentiation of *Dendrobium* species is essential to ensure their quality and safe use, and to preserve *Dendrobium* germplasm resources. From then on, the ITS region sequences are the most popular for evaluating molecular evolution and inter specific level divergences in plants. The ITS regions of 13 *Dendrobium* species exist their unique ITS sequences, therefore, ITS can be used as a characteristic fragment to distinguish *Dendrobium* species. To our knowledge, this is the first report that the ITS region sequences have been applied for molecular identification of wild and cultivated *Dendrobium* species of sections *Formosae* and *Chrysotoxae*.

This study showed that the rDNA ITS sequences in the *Dendrobium* species are powerful for phylogenetic analysis of rapid molecular identification and differentiation to establish the genetic relationships among the wild and cultivated plant belonging to sections *Formosae* and *Chrysotoxae*. Both the genetic distance and phylogenetic analysis demonstrated the rDNA gene in *Dendrobium* species of section *Formosae* was more similar than that in section *Chrysotoxae*. Phylogenesis classification of *Dendrobium* species in section *Formosae* is more close to the finding of morphological observation. The secondary structure of the ITS2 regions could enhanced the clustering patterns of the taxonomic levels, and sequence + structure based phylogenetic analysis was more reliable for the molecular authentication and differentiation of the *Dendrobium* species.

In summary, this study demonstrated that the ITS region sequence analysis is simple, quick, and highly reliable that can be used as an effective tool for identification and classification of *Dendrobium* species, and for of the genus *Dendrobium*. It also provided much useful genetic information about *Dendrobium* species for highly-effective germplasm management and resource protection. In the future, more *Dendrobium* species would be added to verify the findings.

## Abbreviations

ITS: internal transcribed spacer
TCM: traditional Chinese medicine
CTAB: cetyltrimethylammonium bromide
PCR: polymerase chain reaction
NJ: neighbor-joining
MP: maximum parsimony
GC: guanine and cytosine

## References

[CR2] Chen ND, Chen H, Li J, Sang MM, Ding S, Yu H (2015). Discrimination and similarity evaluation of tissue-cultured and wild *Dendrobium* species using Fourier transform infrared spectroscopy. J Mol Struct.

[CR3] Chen ND, You T, Li J, Bai LT, Hao JW, Xu XY (2016). A comparative study of three tissue-cultured *Dendrobium* species and their wild correspondences by headspace gas chromatography-mass spectrometry combined with chemometric methods. J Food Drug Anal.

[CR4] Chiang CH, Yu TA, Lo SF, Kuo CL, Peng WH, Tsay HS (2012). Molecular authentication of *Dendrobium* species by multiplex polymerase chain reaction and amplification refractory mutation system analysis. J Am Soc Hort Sci.

[CR5] Chinese Pharmacopoeia Committee (2015). Pharmacopoeia of The people’s Republic of China.

[CR6] Coleman AW (2003). ITS2 is a double-edged tool for eukaryote evolutionary comparisons. Trends Genet.

[CR9] Feng SG, Jiang Y, Wang S, Jiang MY, Chen Z, Ying QC, Wang HZ (2015). Molecular identification of *Dendrobium* species (*Orchidaceae*) based on the DNA barcode ITS2 region and its application for phylogenetic study. Int J Mol Sci.

[CR10] Ge JC, Zha XQ, Nie CY, Yu NJ, Li QM, Peng DY, Duan J, Pan LH, Luo JP (2018). Polysaccharides from *Dendrobium* huoshanense stems alleviates lung inflammation in cigarette smoke-induced mice. Carbohyd Polym.

[CR13] Kanlayavattanakul M, Lourith N, Chaikul P (2018). Biological activity and phytochemical profiles of *Dendrobium*: a new source for specialty cosmetic materials Author links open overlay panel. Ind Crop Prod.

[CR15] Liang YZ, Xie P, Chan K (2004). Quality control of herbal medicines. J Chromatogr B.

[CR17] Niu ZT, Pan JJ, Xue QY, Zhu SY, Liu W, Ding XY (2018). Plastome-wide comparison reveals new SNV resources for the authentication of *Dendrobium* huoshanense and its corresponding medicinal slice (Huoshan Fengdou). Acta Pharmaceut Sinica B.

[CR19] Sobrino-Gregorio L, Vilanova S, Prohens J, Escriche I (2019). Detection of honey adulteration by conventional and real-time PCR. Food Control.

[CR21] Teixeira da Silva JA, Jin XH, Dobránszki J, Lu JJ, Wang HZ, Zotz G, Cardoso JC, Zeng SJ (2016). Advances in *Dendrobium* molecular research: applications in genetic variation, identification and breeding. Mol Phylogenet Evol.

[CR22] Tsai CC, Peng CI, Huang SC, Huang PL, Chou CH (2004). Determination of the genetic relationship of *Dendrobium* species (*Orchidaceae*) in Taiwan based on the sequence of the internal transcribed spacer of ribosomal DNA. Sci Horticulturae.

[CR23] Wilson JJ, Sing KW, Jaturas N (2019). DNA barcoding: bioinformatics workflows for beginners. Encyclopedia Bioinf Computat Biol.

[CR24] World Checklist of Selected Plant Families; 2015. <http://apps.kew.org/wcsp/home. Accessed 10 Oct 2015

[CR25] Wu SJ, Liu YS, Chen TW, Ng CC, Tzeng WS, Shyu YT (2009). Differentiation of medicinal *Dendrobium* species (*Orchidaceae*) using molecular markers and scanning electron microscopy. J Food Drug Anal.

[CR27] Xiang XG, Schuiteman A, Li DZ, Huang WC, Chung SW, Li JW, Zhou HL, Jin WT, Lai YJ, Li ZY, Jin XH (2013). Molecular systematics of *Dendrobium* (*Orchidaceae*, *Dendrobieae*) from mainland Asia based on plastid and nuclear sequences. Mol Phylogenet Evol.

[CR30] Xu Q, Zhang GQ, Liu ZJ, Luo YB (2014). Two new species of *Dendrobium* (*Orchidaceae: Epidendroideae*) from China: evidence from morphology and DNA. Phytotaxa.

[CR32] Ye Z, Lv Y, Wang ZT, Xu H, Hu ZB (2014). Identification of *Dendrobii caulis* basing on ITS sequence. China J Chin Mater Med.

[CR39] Zeng SJ, Hu SH (2004). The dendrobium orchids.

[CR34] Zha XQ, Luo JP, Wei P (2009). Identification and classification of *Dendrobium* candidum species by fingerprint technology with capillary electrophoresis. S Afr J Bot.

[CR35] Zha XQ, Deng YY, Li XL, Wang JF, Pan LH, Luo JP (2017). The core structure of a Dendrobium huoshanense polysaccharide required for the inhibition of human lens epithelial cell apoptosis. Carbohyd Polym.

[CR36] Zhao XY, Dou MM, Zhang ZH, Zhang DD, Huang CZ (2017). Protective effect of *Dendrobium officinale* polysaccharides on H_2_O_2_-induced injury in H9c2 cardiomyocytes. Biomed Pharmacother.

[CR38] Zhu GH, Ji ZH, Wood JJ, Wood HP (2009). Flora of China.

